# Dynamic changes in peripheral lymphocytes and antibody response following a third dose of SARS-CoV-2 mRNA-BNT162b2 vaccine in cancer patients

**DOI:** 10.1038/s41598-022-25558-8

**Published:** 2022-12-19

**Authors:** Enzo Maria Ruggeri, Fabrizio Nelli, Diana Giannarelli, Agnese Fabbri, Julio Rodrigo Giron Berrios, Antonella Virtuoso, Eleonora Marrucci, Marco Mazzotta, Marta Schirripa, Carlo Signorelli, Mario Giovanni Chilelli, Francesca Primi, Cristina Fiore, Valentina Panichi, Giuseppe Topini, Maria Assunta Silvestri

**Affiliations:** 1 Medical Oncology Unit, Department of Oncology and Hematology, Central Hospital of Belcolle, Strada Sammartinese Snc, 01100 Viterbo, Italy; 2 Biostatistics Unit, Scientific Directorate, IRCCS, Fondazione Policlinico Universitario A. Gemelli, Rome, Italy; 3 Cytofluorimetry Unit, Department of Oncology and Hematology, Central Hospital of Belcolle, Viterbo, Italy; 4 Department of Oncology and Hematology, Microbiology and Virology Unit, Central Hospital of Belcolle, Viterbo, Italy

**Keywords:** RNA vaccines, Cancer therapy, Lymphocytes, Antibodies, Viral infection

## Abstract

The aim of this study was to evaluate the association of circulating lymphocytes profiling with antibody response in cancer patients receiving the third dose of COVID-19 mRNA-BNT162b2 vaccine. Immunophenotyping of peripheral blood was used to determine absolute counts of lymphocyte subsets, alongside detection of IgG antibodies against receptor-binding-domain (RBD) of the SARS-CoV-2 Spike protein (S1) before booster dosing (timepoint-1) and four weeks afterward (timepoint-2). An IgG titer ≥ 50 AU/mL defined a positive seroconversion response. An IgG titer ≥ 4446 AU/mL was assumed as a correlate of 50% vaccine efficacy against symptomatic infections. A total of 258 patients on active treatment within the previous six months were enrolled between September 23 and October 7, 2021. The third dose resulted in an exponential increase in median anti-RBD-S1 IgG titer (*P* < 0.001), seroconversion rates (*P* < 0.001), and 50% vaccine efficacy rates (*P* < 0.001). According to ROC curve analysis, T helper and B cells were significantly associated with seroconversion responses at timepoint-1, whereas only B cells were relevant to 50% vaccine efficacy rates at timepoint-2. A positive linear correlation was shown between anti-RBD-S1 IgG titers and these lymphocyte subset counts. Multivariate analysis ruled out a potential role of T helper cells but confirmed a significant interaction between higher B cell levels and improved antibody response. These findings suggest that peripheral counts of B cells correlate with humoral response to the third dose of mRNA-BNT162b2 vaccine in actively treated cancer patients and could provide insights into a more comprehensive assessment of vaccination efficacy.

## Introduction

The vaccination coverage against COVID-19 pandemic prioritized cancer patients on active treatment because of the increased morbidity and mortality rates associated with this immunocompromising condition^1^. Even without the evidence of randomized controlled trials, several observational studies have consistently indicated that the two-dose schedule of mRNA-based vaccines is safe and effective in recipients with solid malignancies^2^.

The waning in antibody titers within six months of the second dose of mRNA-BNT162b2 (tozinameran) vaccine^3,4^, as well as the loss of neutralizing activity of vaccine-induced humoral response against SARS-CoV-2 variants of concern (VOC), renewed concerns in the oncology community^5,6^. Subsequent evidence of breakthrough infections in fully vaccinated cancer patients suggested the need for additional interventions to maintain adequate immunity and biomarkers of clinical protection from symptomatic COVID-19^7^. Regarding the first issue, after demonstrating efficacy in solid organ transplant recipients^8,9^, regulatory agencies in North America and Europe recommend a third homologous dose (or booster) of mRNA-based vaccine for immunocompromised patients, including those on active cancer treatments. The paucity of data on its immunogenicity is inconsistent with the rapid deployment of this preventive measure^10^. The availability of biomarkers predicting the clinical efficacy of vaccination is also a controversial issue ^11^. Most of the research focused on determining an optimal antibody titer threshold, as higher levels have been thought to be associated with neutralization capacity and lower risk of symptomatic infection^12,13^. Nevertheless, a reliable correlate of humoral response for this purpose remains ill-defined^14^. In this context, the vaccine-induced cell-mediated immune response has been considered essential for humoral immunity and clinical protection even in high-risk conditions^15,16^. However, its role has been less thoroughly characterized, with few studies investigating SARS-CoV-2-specific T and B cell responses after full-dose vaccination^17–20^. More recent data suggest that absolute counts of circulating lymphocyte subsets were associated with vaccine response in a highly vulnerable population receiving CD20 B-cell depletion treatments^21,22^, but their role has never been investigated in patients with solid tumors on active systemic therapies.

To address these issues, the Vax-On-III-Profile study prospectively assessed the potential association of circulating lymphocyte subset counts with antibody response and vaccine efficacy correlates in actively treated cancer patients receiving the third dose of tozinameran.

## Methods

### Design and participants

The Vax-On-III-Profile was a prospective, single-center, observational cohort study. The referring Ethics Committee approved this experimental project (Comitato Etico Lazio 1, Rome, Italy; protocol Number: 1407/CE Lazio1). The study protocol adheres to the reporting guidelines for Strengthening the Reporting of Observational Studies in Epidemiology (STROBE) and received formal registration (clinical trial identifier: EudraCT Number 2021-002,611-54). The main eligibility requirements included age ≥ 18 years, histological diagnosis of solid tumor, active oncologic treatment underway or completed within the previous six months, and fulfillment of the two-dose series of tozinameran at least 22 weeks before enrollment. The exclusion criteria comprised life expectancy < 12 weeks, active and concomitant hematological malignancy, documented COVID-19 infection at any time, and pregnancy. Eligible patients received 30 μg of tozinameran intramuscularly at least 24 h apart from their expected anticancer treatment (timepoint-1). Blood samples for serological and immunological testing were collected at timepoint-1 and four weeks afterward (timepoint-2). Adherence to vaccination was voluntary and carried out after the acquisition of institutional informed consent. All participants gave specific written informed consent for the study proposal and procedures, but their participation was not a prerequisite for receiving the booster dose. The methods used in this study comply with the tenets of the 1964 Declaration of Helsinki and its subsequent amendments, and were performed in accordance with the relevant guidelines and regulations.

The first co-primary endpoint was the correlation of dynamic changes in circulating lymphocyte counts with IgG antibody titers against the receptor-binding domain (RBD) of the SARS-CoV-2 Spike protein (S1) and seroconversion responses. The second co-primary endpoint was to determine whether lymphocyte subpopulation dynamics could show an independent interaction with antibody and seroconversion responses. As of November 10, 2021, the data would be considered conclusive.

### Serological test

Whole blood samples were collected (3 mL/subject in BD Vacutainer Plus Plastic Serum Tubes) and separated by immediate centrifugation. Serum specimens were analyzed at each sampling. The SARS-CoV-2 IgG II Quant assay on the ARCHITECT i2000sr automated platform (Abbott Laboratories, Diagnostics Division, Sligo, Ireland) was used to detect anti-RBD-S1 IgG antibodies according to the manufacturer's instructions^23^. The results were reported as arbitrary units (AU)/mL, with a cut-point ≥ 50 AU/mL indicating a positive seroconversion response. In addition, an anti-RBD-S1 antibody titer ≥ 4446 AU/mL was assumed as a reliable threshold associated with 50% vaccine efficacy against a symptomatic COVID-19 infection. Since the study by Feng et al.^12^ from which we derived this correlate employed a different immunoassay, we did not perform any direct assessment of the clinical efficacy of the third dose of tozinameran as related to this parameter. On the basis of adequate correlation with the serological test in our study^24^, we instead chose this threshold because it was considered an immune response more suitable than the seroconversion with respect to antibody titer levels induced by booster dosing.

### Immunophenotyping

Whole blood draws for flow cytometry analysis (3 mL/subject in BD Vacutainer spray-coated K2EDTA Tubes) were collected at the same time as sampling for serological testing. The panel for staining included the following monoclonal antibodies: CD3 FITC, CD4 PE-Cy7, CD8 APC-Cy7, CD19 APC, CD45 PerCP-Cy5.5, and CD16 PE + CD56 PE; BD Biosciences, San Jose, CA). As we have previously described^25^, the BD Multitest 6-color TBNK reagent was used to determine absolute counts of B and NK cells, as well as CD4 and CD8 subpopulations of T cells. The data were collected using the BD FACSCanto II system and BD FACSCanto clinical software (BD Biosciences, San Jose, CA) as directed by the manufacturer^26^.The results were reported as absolute cell counts/µL for each lymphocyte subset. An example of the gating strategy is shown in Supplementary Fig. 1.

### Statistical analysis

Mean with Standard Deviation (SD) was used to describe normally distributed variables, while Median with 95% Confidence Intervals (CI) or Interquartile Range (IQR) was reported for skewed variables. Comparative assessments were performed by applying Pearson's *χ*^*2*^ test for categorical data and Mann–Whitney *U* test for continuous variables. The Wilcoxon signed-rank and McNemar tests were applied for pairwise comparisons. A preliminary multivariate analysis was performed by adjusting a generalized linear model on the logarithmic (log) values of each subset of lymphocytes as a function of predefined covariates, including sex, age, Eastern Cooperative Oncology Group Performance Status (ECOG PS), treatment setting, corticosteroid therapy, type and timing of active treatments. At both time points, receiver operating characteristic (ROC) curves were calculated to determine the sensitivity and specificity of peripheral blood lymphocyte subsets in relation to positive seroconversion responses. Variables with a statistically significant association to the intended outcome were considered relevant for subsequent evaluations. The Youden index was applied to determine the optimal cut-point. The Spearman method was used to assess the correlation between the log values anti-RBD-S1 IgG titers and significant lymphocyte subset counts. A secondary multivariate analysis was performed by fitting the same model to anti-RBD-S1 IgG log titers and seroconversion responses or 50% vaccine efficacy rates as a function of circulating lymphocyte levels in addition to independent covariates described above. All tests performed were two-sided and a *P* value < 0.05 was considered significant. SPSS (IBM SPSS Statistics for Windows, version 23.0, Armonk, NY) and Prism (GraphPad, version 9) software were used for statistical evaluations and figure rendering, respectively.

## Results

### Patient characteristics

This study enrolled 258 consecutive patients who met the inclusion criteria and received a third dose of tozinameran between September 23 and October 7, 2021. The entire population consisted of 202 (78.3%) patients who had previously participated in the Vax-On study^27^ and 56 (21.7%) cases who were recruited at a later time point. The patients’ flow diagram depicts the reasons why a portion of the original study population did not receive the third dose of tozinameran (Supplementary Fig. 2). A total of 198 patients (76.7%) were on active treatment, while the remaining 60 cases (23.3%) had discontinued it by at least 28 days. Most patients were female (58.1%), had ECOG PS 0-1 (94.6%) and metastatic disease (72.1%). Cytotoxic chemotherapy (37.2%) and targeted therapy (35.6%) were the most frequent active treatments. Table 1 details the baseline characteristics of enrolled patients.

**Table 1:** Demographic and clinical characteristics of the study population.

Characteristics	All patients, N = 258 (100%)
Mean age, years (SD)	64.6 (10.6)
**Sex**	
Female	150 (58.1%)
Male	108 (41.9%)
**ECOG PS**	
0	129 (50%)
1	115 (44.6%)
2	14 (5.4%)
**Tumor type**	
Breast	83 (32.1%)
Lung	50 (19.4%)
Kidney	9 (3.5%)
Prostate	5 (1.9%)
Colorectal	51 (19.8%)
Urothelial	11 (4.3%)
Pancreatic	6 (2.3%)
Gastric	11 (4.3%)
Skin (Melanoma, Merkel-cell)	5 (1.9%)
Gynaecological	8 (3.1%)
Head and Neck	3 (1.2%)
Brain	6 (2.3%)
Other^a^	10 (3.9)
**Extent of disease**	
Local	72 (27.9%)
Metastatic	186 (72.1%)
**Type of last active treatment**	
Cytotoxic chemotherapy	96 (37.2%)
Targeted therapy	92 (35.6%)
Immune checkpoint inhibitors	30 (11.6%)
Hormonal therapy	13 (5%)
Cytotoxic chemotherapy + biological therapy	18 (6.9%)
**Treatment setting**	
Adjuvant or neoadjuvant	80 (31%)
Metastatic, first line	126 (48.9%)
Metastatic, second or later line	52 (20.1%)
**Time from last active treatment**	
> 28 days	60 (23.3%)
≤ 28 days	198 (76.7%)
**Reasons for discontinuation of active treatment**	
Treatment completion	52 (20.1%)
Disease progression	6 (2.3)
Refusal to continue	1 (0.4%)
Other reasons	1 (0.4%)
Time (days) from the first dose of vaccine, median (IQR)	185 (167–197)
Time (days) from the second dose of vaccine, median (IQR)	164 (149–177)
Corticosteroid therapy^b^	43 (17%)
G-CSF therapy^c^	8 (3%)

*SD* standard deviation, *ECOG PS* Eastern Cooperative Oncology Group Performance Status, *IQR* interquartile range, *G-CSF* granulocyte-colony stimulating factor, *NA* not applicable. ^a^Other cancer types included soft-tissue sarcoma, thymoma, testicular cancer, hepatobiliary cancer, esophageal cancer, and GIST; ^b^corticosteroid therapy indicates ≥ 10 mg per day of prednisone or equivalent for at least 7 days in the 28 days preceding third vaccine dose; ^c^G-CSF therapy indicates administration of granulocyte colony-stimulating factor in the 28 days preceding third vaccine dose.

### Immunogenicity and efficacy

Serological testing was performed in all patients at timepoint-1 and in 253 (98.1%) patients at timepoint-2, with reasons for missing assessment shown in Supplementary Fig. 2. The third dose of vaccine resulted in an exponential increase in median anti-RBD-S1 IgG titer [from 419 AU/mL (95% CI 345–505) to 14,223 AU/mL (95% CI 12,104–16,363), *P* < 0.001], seroconversion rates (from 89.5 to 98.8%, *P* < 0.001), and 50% vaccine efficacy rates (from 5 to 76.7%, *P* < 0.001). After a median follow-up of 145 days (IQR 140–153), 10 of the study participants (3.8%) reported contracting SARS-CoV-2 infection, none of which was clinically severe.

### Dynamic changes in peripheral lymphocyte counts

**Figure 1:**
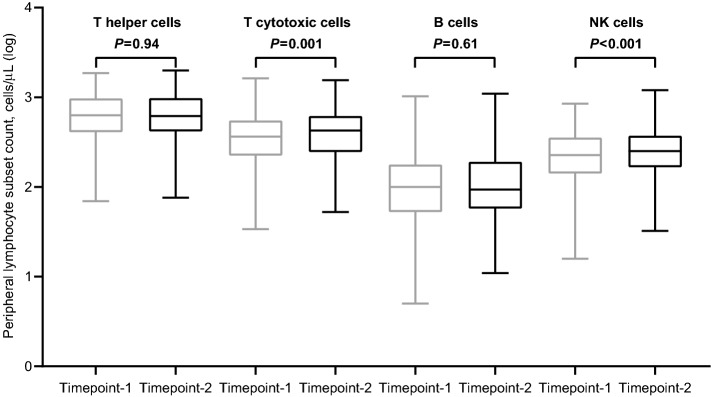
Dynamic changes in peripheral lymphocyte subpopulation counts. Bars represent median values with 95% confidence intervals. Differences between groups were assessed using the Wilcoxon signed-rank test. A two-sided *P* value of < 0.05 was considered statistically significant. Log, logarithmic; T helper cells, CD3 + CD4 + cells; T cytotoxic cell, CD3 + CD8 + ; B cells, CD19 + ; NK, Natural killer, CD16 + CD56 + . Timepoint-1 denotes assessment before the third dose of tozinameran; Timepoint-2 denotes assessment four weeks after the third dose of tozinameran.

Peripheral blood immunophenotyping was completed in 256 (99.2%) and 251 (97.2%) patients at timepoint-1 and -2, respectively (Supplementary Fig. 2). We observed a considerable variability in the values of each subset of lymphocytes among patients, which even increased after the booster dose. Compared with baseline, the third dose of tozinameran did increase the median values of all lymphocyte subsets. This difference was statistically significant only for T cytotoxic (*P* = 0.001) and NK cells (*P* < 0.001, Fig. 1). In view of their potential effect on lymphocyte composition, we performed the same comparative evaluation according to the different types of active treatments and corticosteroid therapy. The incremental variation induced by the booster dose was confirmed in all subgroups. Of note, an increase in both T cytotoxic and NK cells was significant only in patients receiving cytotoxic chemotherapy or its combination with biological agents (Supplementary Fig. 3). The same result was also found in patients who were not being treated with corticosteroids at immunosuppressive dosage levels before the third dose of tozinameran (Supplementary Fig. 4). We also performed a preliminary multivariate analysis to verify the effects of independent clinical variables on lymphocyte subset counts at timepoint-1. Immunosuppressive corticosteroid therapy before the third dose correlated with lower counts in T helper (*P* < 0.001) and B cells (*P* = 0.017), whereas no significant interaction was found with types or timing of active treatments (Supplementary Table 1).

### ROC curves and correlation analysis

**Figure 2:**
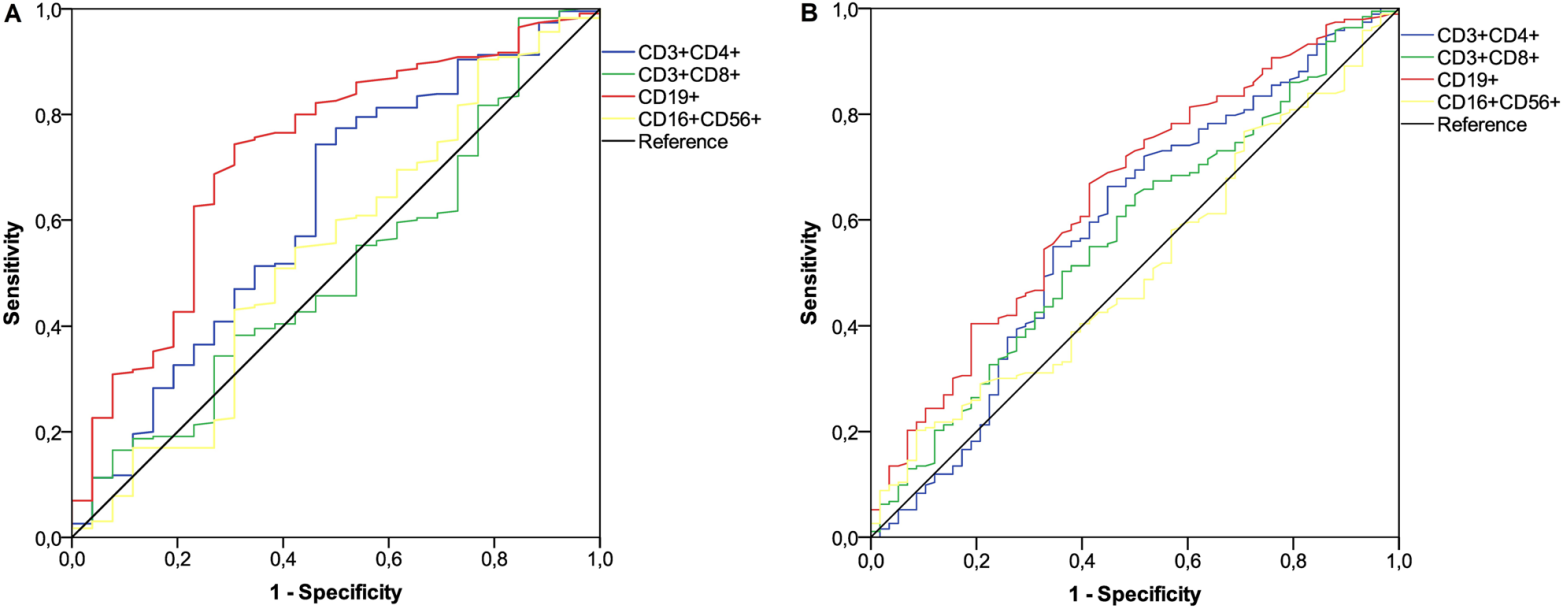
ROC curve analysis. Receiver operating characteristic (ROC) curves are represented for subsets of peripheral lymphocytes. (**A**) ROC analysis showing the performance of absolute counts of peripheral lymphocyte subsets in distinguishing positive seroconversion responses at timepoint-1. AUC for the subpopulation relative values: T helper cells (CD3 + CD4 +): 0.62 (95% CI 0.49–0.74; *P* = 0.044); T cytotoxic cells (CD3 + CD8 +): 0.50 (95% CI 0.39–0.62; *P* = 0.89); B cells (CD19 +): 0.72 (95% CI 0.61–0.83; *P* < 0.041); NK cells (CD16 + CD56 +): 0.53 (95% CI 0.41–0.66; *P* = 0.52). (**B**) ROC analysis showing the performance of absolute counts of peripheral lymphocyte subsets in distinguishing 50% vaccine efficacy rates at timepoint-2; AUC for the subpopulation relative values: T helper cells (CD3 + CD4 +): 0.58 (95% CI 0.49–0.67; *P* = 0.05); T cytotoxic cells (CD3 + CD8 +): 0.57 (95% CI 0.48–0.65; *P* = 0.09); B cells (CD19 +): 0.64 (95% CI 0.56–0.72; *P* = 0.001); NK cells (CD16 + CD56 +): 0.51 (95% CI 0.42–0.59; *P* = 0.79). AUC, area under the curve; NK, Natural Killer; CI, confidence interval. Timepoint-1 denotes assessment before the third dose of tozinameran; timepoint-2 denotes assessment four weeks after the third dose of tozinameran; an antibody titer ≥ 50 AU/mL indicates a positive seroconversion response; antibody titer ≥ 4446 AU/ml was associated with 50% vaccine efficacy against a symptomatic COVID-19 infection.

**Figure 3:**
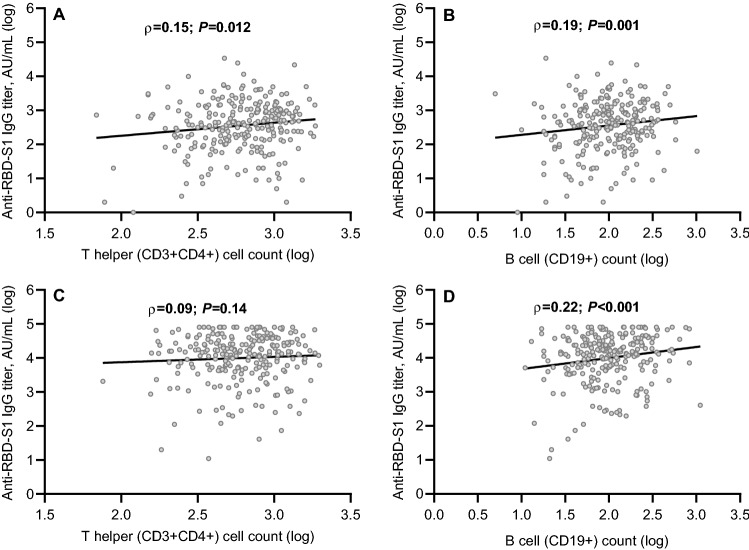
Correlation between antibody titers and lymphocyte subpopulation counts. (**A**) T helper cell (CD3 + CD4 +) counts before the third dose of tozinameran. (**B**) B cell (CD19 +) counts before the third dose of tozinameran. (**C**) T helper cell counts after the third dose of tozinameran. (**D**) B cell counts after the third dose of tozinameran. Correlation was assessed with the Spearman’s test; a two-sided *P* value < 0.05 was considered statistically significant. RBD-S1, receptor-binding domain (RBD) of the SARS-CoV-2 Spike protein (S1); AU, Arbitrary Unit; log, logarithmic values.

An initial ROC curve was calculated to establish the relationship between lymphocyte subset counts before booster dosing and seroconversion responses at timepoint-1. T helper and B cell subpopulations showed a significant association and were found to be valuable in predicting the likelihood of a positive outcome (Fig. 2A). With a cut-off value of 441/µL for T helper and 58/µL for B cells, the sensitivity and specificity were 0.74 and 0.54, and 0.76 and 0.62, respectively. Evaluating anti-RBD-S1 log IgG titers as a function of these lymphocyte log counts demonstrated a significant positive linear correlation for either T helper [*ρ* = 0.15 (95% CI 0.02–0.27), *P* = 0.012; Fig. 3A] and B cell subpopulation [*ρ* = 0.19 (95% CI 0.06–0.33), *P* = 0.001; Fig. 3B]. Given that almost all patients achieved a seroconversion response at timepoint-2, a subsequent ROC curve analysis was computed to determine the relationship between lymphocyte subset counts after booster and 50% vaccine efficacy at the same time point. Only the subset of B cells revealed a significant association and was therefore deemed relevant for subsequent evaluations (Fig. 2B). The cut-point value of 77/µL defined sensitivity and specificity levels of 0.68 and 0.57, respectively. In contrast to the findings for T cell log counts [*ρ* = 0.09 (95% CI -0.04 -0.21), *P* = 0.14; Fig. 3C], a significant positive linear correlation was confirmed between anti-RBD-S1 log IgG titers and B cell log counts at timepoint-2 [*ρ* = 0.22 (95% CI 0.09–0.34), *P* < 0.001; Fig. 3D]. We also performed the same correlation analysis in subgroups of patients featuring the same active treatment at both time points (Supplementary Figs. 5 to 10). It is worth noting that the B lymphocyte subpopulation showed higher coefficients at timepoint-1 and/or 2, confirming previous results.

### Analysis of antibody response

**Figure 4:**
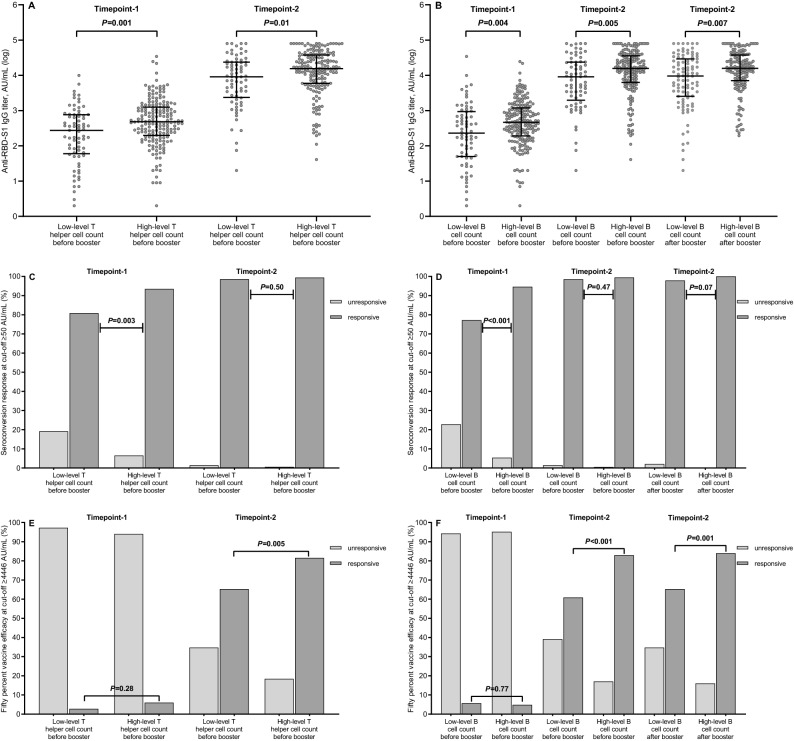
Antibody response by level of lymphocyte subpopulation counts. (**A**) Comparison of scatter plot distributions and medians of antibody titers by level of T helper cell counts. (**B**) Comparison of scatter plot distributions and medians of antibody titers by level of B cell counts. (**C**) Comparison of seroconversion response rates by level of T helper cell counts. (**D**) Comparison of seroconversion response rates by level of B cell counts. (**E**) Comparison of 50% vaccine efficacy rates by level of T helper cell counts. (**F**) Comparison of 50% vaccine efficacy rates by level of B cell counts. Bars represent median values with 95% Confidence Intervals. Differences between groups were assessed using the Mann–Whitney *U* test (continuous variable) or the Pearson's *χ*^*2*^ test (categorical variables), as appropriate. A two-sided *P* value of < 0.05 was considered statistically significant. RBD-S1, receptor-binding domain (RBD) of the SARS-CoV-2 Spike protein (S1); AU, Arbitrary Unit; log, logarithmic values. Timepoint-1 denotes assessment before the third dose of tozinameran; Timepoint-2 denotes assessment four weeks after the third dose of tozinameran; an antibody titer ≥ 50 AU/mL indicates a positive seroconversion response; antibody titer ≥ 4446 AU/ml was associated with 50% vaccine efficacy against a symptomatic COVID-19 infection.

ROC curve analyses established cut-point values that allowed the subpopulations of T helper and B cells to be divided into low- and high-level subgroups. On a univariate comparison, both high-level T helper and B cell counts before the third dose of vaccine resulted in a significant improvement of antibody titers, seroconversion rates, and 50% vaccine efficacy rates at timepoint-2. After booster dosing, the high-level B cell subset also had significantly increased antibody titers and 50% vaccine efficacy rates (Table 2 and Fig. 4). All breakthrough infections occurred in the subgroup of patients with low-level B cell counts at both time points (*P* < 0.001).

**Table 2:** Univariate analysis of antibody response.

	Timepoint-1							Timepoint-2				
	Evaluable patients, N = 256 (100%)	Median IgG titer, AU/mL, (95% CI)	*P* value	Seroconversion response^a^, N (%)	*P* value	50% vaccine efficacy^b^, N (%)	*P* value	Evaluable patients, N = 251 (100%)	Median IgG titer, AU/mL, (95% CI)	*P* value	50% vaccine efficacy^b^, N (%)	*P* value
**T helper cell count before the third dose of vaccine**												
Low level (≤ 441/µL)	73 (28.5)	275 (155–379)	**0.001**	59 (80.8)	**0.003**	2 (2.7)	0.28	72 (28.7)	9097 (5617–14,952)	**0.01**	47 (65.3)	**0.005**
High level (> 441/µL)	183 (71.5)	483 (408–665)		171 (93.4)		11 (6.0)		179 (71.3)	15,507 (13,248–17,898)		146 (81.6)	
**B cell count before the third dose of vaccine**												
Low level (≤ 58/µL)	70 (27.3)	230 (150–421)	**0.004**	54 (77.1)	** < 0.001**	4 (5.7)	0.77	69 (27.5)	9080 (4251–12,456)	**0.005**	42 (60.9)	** < 0.001**
High level (> 58/µL)	186 (72.7)	465 (371–599)		176 (94.6)		9 (4.8)		182 (72.5)	15,720 (13,248–17,897)		151 (83.0)	
**B cell count after the third dose of vaccine**												
Low level (≤ 77/µL)	–	–	–	–	–	–	–	95 (37.8)	9566 (5396–14,942)	**0.007**	62 (65.3)	**0.001**
High level (> 77/µL)	–	–	–	–	–	–	–	156 (62.2)	15,860 (13,586–18,816)		131 (84.0)	

Statistically significant *P* values are highlighted in bold. Differences between groups were assessed using the Mann–Whitney *U* test (continuous variable) or the Pearson's *χ*^*2*^ test (categorical variables), as appropriate. A two-sided *P* value of < 0.05 was considered statistically significant. IgG, immunoglobulin G; AU, arbitrary unit; CI, confidence intervals. ^a^Seroconversion response at cut-off ≥ 50 AU/mL; ^b^50% vaccine efficacy at cut-off ≥ 4446 AU/mL; Timepoint-1 denotes antibody response assessment six months after starting vaccination; Timepoint-2 denotes antibody response assessment four weeks after the third dose of tozinameran.

The subgroups defined by T helper and B cell levels at timepoint-1, as well as B cell levels at timepoint-2, were included in the multivariate testing as independent covariates. Different T helper cell levels at timepoint-1 were not found to be associated with any of the immune parameters. High-level B cell counts before the third dose of vaccine were confirmed to be significantly correlated with both an improved humoral response before the booster dose (*P* = 0.047) and increased 50% vaccine efficacy rates at timepoint-2 (*P* = 0.007). High-level B cell counts after the booster dose resulted in a significant interaction with either increased antibody titers (*P* = 0.01) and 50% vaccine efficacy rates at timepoint-2 (*P* = 0.003). While types and timing of active treatments did not show a reliable influence, immunosuppressive corticosteroid therapy and ECOG PS2 had detrimental effects on antibody responses at both time points (Table 3).

**Table 3:** Multivariate analysis of antibody response.

Covariates	Timepoint-1						Timepoint-2			
	Anti-RDB S1 IgG titer (log)		Seroconversion response^b^		50% vaccine efficacy^c^		Anti-RDB S1 IgG titer (log)		50% vaccine efficacy^c^	
	Beta (95% CI)	*P* value	Beta (95% CI)	*P* value	Beta (95% CI)	*P* value	Beta (95% CI)	*P* value	Beta (95% CI)	*P* value
**Sex**										
Male vs. female	− 0.24 (− 0.30 to 0.05)	0.17	0.07 (− 0.96 to 1.10)	0.88	0.87 (− 0.49 to 2.25)	0.21	− 0.14 (− 0.32 to 0.04)	0.13	− 0.72 (− 1.41 to − 0.03)	**0.041**
**Age (years)**										
> 55 vs. ≤ 55	0.01 (− 0.19 to 0.21)	0.90	− 0.79 (− 2.26 to 0.66)	0.28	0.64 (1.15 to 2.44)	0.48	0.08 (− 0.12 to 0.28)	0.44	0.23 (− 0.58 to 1.05)	0.57
**ECOG PS**										
0^a^	–	–	–	–	–	–	–	–	–	–
1	− 0.03 (− 0.21 to 0.14)	0.71	0.69 (− 0.42 to 1.81)	0.22	− 0.54 (− 2.00 to 0.91)	0.46	− 0.08 (− 0.26 to 0.10)	0.38	0.08 (− 0.63 to 0.80)	0.81
2	− 0.50 (− 0.89 to -0.11)	**0.011**	− 1.24 (− 3.02 to 0.53)	0.17	− 20.21 (NA)	0.99	− 0.52 (− 0.91 to − 0.13)	**0.009**	− 1.35 (− 2.69 to − 0.02)	**0.045**
**Treatment setting**										
Adjuvant or neoadjuvant^a^	–	–	–	–	–	–	–	–	–	–
Metastatic, first line	0.03 (− 0.17 to 0.25)	0.71	− 0.09 (− 1.41 to 1.22)	0.89	− 0.23 (− 1.80 to 1.34)	0.77	0.02 (− 0.18 to 0.24)	0.80	0.25 (− 0.60 to 1.11)	0.56
Metastatic, second or later line	− 0.07 (− 0.33 to 0.17)	0.55	− 0.87 (− 2.41 to 0.65)	0.26	0.22 (− 1.61 to 2.07)	0.80	− 0.08 (− 0.34 to 0.16)	0.49	− 0.43 (− 1.43 to 0.56)	0.39
**Corticosteroid therapy**										
Yes vs. no	− 0.51 (− 0.74 to − 0.27)	**0.001**	− 1.97 (− 3.05 to − 0.90)	** < 0.001**	− 0.68 (− 2.95 to 1.59)	0.55	− 0.24 (− 0.48 to − 0.01)	**0.041**	− 0.26 (− 1.10 to 0.57)	0.53
**Timing of active**										
Treatment ≤ 28 days vs. > 28 days	0.32 (0.08 to 0.56)	**0.008**	1.24 (− 0.09 to 2.58)	0.07	20.77 (NA)	0.99	0.14 (− 0.10 to 0.38)	0.25	0.21 (− 0.70 to 1.13)	0.64
**Last active treatment**										
Targeted therapy^a^	–	–	–	–	–	–	–	–	–	–
Cytotoxic chemotherapy	0.08 (− 0.13 to 0.30)	0.44	0.93 (− 0.41 to 2.29)	0.17	0.88 (− 0.60 to 2.37)	0.24	0.04 (− 0.18 to 0.26)	0.72	0.06 (− 0.81 to 0.95)	0.87
Immune checkpoint inihibitors	− 0.22 (− 0.52 to 0.07)	0.14	− 0.22 (− 1.86 to 1.41)	0.79	− 19.85 (NA)	0.99	− 0.21 (− 0.51 to 0.08)	0.15	− 0.39 (− 0.81 to 0.95)	0.47
Hormonal therapy	0.14 (− 0.29; 0.58)	0.51	0.31 (− 2.25 to 2.88)	0.80	21.23 (NA)	0.99	0.08 (− 0.35 to 0.52)	0.69	0.94 (− 1.36 to 3.26)	0.42
Chemotherapy & biologics	− 0.36 (− 0.66 to − 0.07)	**0.014**	− 0.81 (− 2.41 to 0.77)	0.31	− 19.74 (NA)	0.99	− 0.26 (− 0.56 to 0.02)	0.07	− 0.64 (− 1.74 to 0.46)	0.25
**T helper cell level before booster**										
High vs. low	0.15 (− 0.06 to 0.36)	0.16	0.47 (− 0.65 to 1.60)	0.41	1.13 (− 0.85 to 3.11)	0.26	0.05 (− 0.15 to 0.26)	0.59	0.27 (− 0.50 to 1.05)	0.48
**B cell level before booster**										
High vs. low	0.20 (0.02 to 0.40)	**0.047**	1.60 (0.51 to 2.69)	**0.004**	− 0.74 (− 2.21 to 0.73)	0.32	0.19 (− 0.01 to 0.39)	0.05	1.00 (0.27 to 1.72)	**0.007**
**B cell level after booster**										
High vs. low	–	–	–	–	–	–	0.22 (0.05 to .040)	**0.01**	0.97 (0.33 to 1.65)	**0.003**

Statistically significant *P* values are highlighted in bold. *P* values derived from parametric 2-sided Wald’s *χ2* test with Bonferroni (α = 0.01) correction for multiple comparisons. A two-sided *P* value of < 0.05 was considered statistically significant. RBD-S1, receptor binding domain of the S1 subunit of the SARS-CoV-2 spike protein; log, logarithmic values; IgG, immunoglobulin G; AU, arbitrary unit; CI, confidence intervals; ECOG PS, Eastern Cooperative Oncology Group Performance Status; NA, not applicable. ^a^Reference category; ^b^Seroconversion response at cut-off ≥ 50 AU/mL; ^c^50% vaccine efficacy response at cut-off ≥ 4446 AU/mL; Timepoint-1 denotes antibody response assessment before the third dose of tozinameran; Timepoint-2 denotes antibody response assessment four weeks after the third dose of tozinameran.

## Discussion

The Vax-On-III-Profile is a longitudinal interventional study investigating the predictive value of dynamic changes in peripheral lymphocyte subsets on humoral response to the third dose of tozinameran. To our knowledge, this is the first study evaluating these biomarkers and vaccine efficacy in patients with solid malignancies receiving active treatments. The third dose of tozinameran induced a 34-fold increase in the median anti-RBD-S1 IgG titer. This change led to an outright improvement in seroconversion rates by 10%, with only a few patients (1.2%) remaining seronegative 28 days after the booster dosing. An antibody response of this magnitude is overlaid by that described in similar studies that employed the same serologic testing methodology^28,29^. This enhanced humoral immunity also appears to be consistent with the results of two studies showing that mRNA-vaccine booster dosing induces a strengthened neutralizing antibody response against VOCs in cancer patients^30,31^. Our additional analysis aimed to overcome the misleading value of the seroconversion response, which was developed as a diagnostic tool for prior SARS-Cov-2 exposure but has never been correlated to a protective clinical effect^32^. The minimum antibody titer required to protect against symptomatic SARS-Cov-2 infection is unknown but presumably higher than the seroconversion cut-off^12^. Assuming that a 50% vaccine efficacy-related antibody titer indicates an intermediate level of clinical protection, the booster dose would have reduced the risk of symptomatic infection in more than 76% of patients.

The positive correlation of baseline B cell counts with anti-RBD-S1 IgG titers at both time points suggests a direct relationship between these immune parameters. The significant association between incremental variations in B lymphocytes and humoral response after booster vaccination further supports the strength of this correlation. A noteworthy observation was the independent interaction between iterative B cell levels and antibody responses on multivariate analysis. These findings suggest the dynamics of B cell counts closely follow changes in humoral immunogenicity after the third dose of tozinameran. The choice of numbers of circulating lymphocyte subpopulations as a correlate of vaccine-induced adaptive immunity might represent a controversial issue. In vaccinated cancer patients, the cell-mediated immune response was characterized through enzyme-linked immune adsorbent spot (ELISpot) assays to quantify interferon-gamma (IFNγ)—producing SARS-CoV-2-specific T cells^17–20,33^ and high-resolution flow cytometry assays incorporating multiple cytokines and activation markers for RBD-S1-specific memory B cell profiling^17^. Although these assays are ideal because of their high sensitivity and specificity, the suboptimal level of procedure standardization and methodological complexities still prevent their widespread use. Immunophenotyping of peripheral blood in this study provides a nonspecific description of lymphocyte response to tozinameran booster vaccination. This approach has inherent strengths and weaknesses. The main advantage of this methodology is the high level of procedure standardization and reproducibility of the results, as it is commonly used for the diagnosis and monitoring of hematologic malignancies and is available in most facilities^34^. However, peripheral lymphocyte counts may be influenced by vaccine-independent variables, the most relevant of which are the effects of different types and timing of cancer treatments^35,36^. Preliminary multivariate analysis ruled out a selection imbalance due to direct interaction with the above factors but showed a significant effect of immunosuppressive corticosteroid dosing on T helper and B cell counts. The unavailability of comparable studies in patients with solid malignancies makes it challenging to determine the clinical significance of our findings. Even so, several studies have found a direct correlation between vaccine-induced absolute T helper and B cell counts and anti-spike IgG antibody titers in patients on CD20 B-cell-depleting treatments^21,22,37^. There was also a high concordance between SARS-CoV-2-specific T and B cell reaction testing and circulating lymphocyte immunophenotyping results. Given the differences among patients with hematologic and solid malignancies, the described experimental evidence is consistent with our findings, confirming the validity of this methodological approach to adaptive immunity associated with tozinameran vaccination.

In addition to the above methodological issues, this study recognizes further shortcomings. The experimental design could not include a control group of age- and sex-matched healthy volunteers since booster vaccination had not yet been licensed for these categories at accrual time. The health contingency accounted for "all-comers" enrollment but did not allow for adequate stratification of participants. This flaw increased the likelihood of selection bias and false-positive results from multivariable statistical comparisons, the significance of which should therefore be considered as hypothesis-generating. The measurement of anti-RBD-S1 IgG titers and assumption of 50% vaccine efficacy relied on the wild-type SARS-CoV-2 strain, which could not account for the high evasive capacity of subsequent Omicron VOC spread^38,39^. In this regard, the use of an assay for neutralizing activity of vaccine-induced antibody levels would have been optimal, but procedural constraints prevented us from implementing this method of investigation^40^. Finally, although we found a significant difference in the clinical outcome of vaccination, the rate of breakthrough infections is still too low (< 4%). This finding may support vaccine efficacy itself or, more likely, reflects the effects of non-pharmaceutical interventions.

## Conclusion

This cohort study confirms the enhanced immunogenicity and, presumably, clinical efficacy of the third dose of tozinameran in an extensive unselected population that may adequately represent cancer patients treated in the real world. Our data suggest that dynamic changes in circulating B lymphocyte counts, as assessed by widely available immunophenotyping of peripheral blood, correlate with the reliability of antibody response. Although unrelated to antibody response, the significant increase in T cytotoxic and NK cells induced by the booster dose deserves further investigation of their potential effects on anticancer treatment outcomes. The limitations described warrant confirmation by independent prospective cohorts. Upon validation and longer follow-up of clinical events, our results could provide insights into a more comprehensive assessment of the efficacy of SARS-CoV-2 vaccination with respect to further active immunization strategies.

## Supplementary Information

Supplementary Information.

## Data Availability

The datasets generated and/or analysed during the current study are available in the figshare repository at http://doi.org/10.6084/m9.figshare.19440062.
