# Supplementary material for: Dynamic changes in peripheral lymphocytes and antibody response following a third dose of SARS-CoV-2 mRNA-BNT162b2 vaccine in cancer patients
Source: Sci Rep. 2022 Dec 19;12:21908. doi: 10.1038/s41598-022-25558-8 (PMC9761743; doi:10.1038/s41598-022-25558-8)
Supplement: Supplementary file 1 — Supplementary Information. [file 41598_2022_25558_MOESM1_ESM.pdf]

Title

Dynamic changes in peripheral lymphocytes and antibody response following a third dose of SARS-CoV-2 mRNA-BNT162b2 vaccine in cancer patients

Authors

Enzo Maria Ruggeri[1*], Fabrizio Nelli[1], Diana Giannarelli[2], Agnese Fabbri[1], Julio Rodrigo Giron Berrios[1], Antonella Virtuoso[1], Eleonora Marrucci[1], Marco Mazzotta[1], Marta Schirripa[1], Carlo Signorelli[1], Mario Giovanni Chilelli[1], Francesca Primi[1], Cristina Fiore[1], Valentina Panichi[3], Giuseppe Topini[3] and Maria Assunta Silvestri[4].

[1] Department of Oncology and Hematology, Medical Oncology Unit, Central Hospital of Belcolle, Viterbo, Italy

[2] Biostatistics Unit, Scientific Directorate, Fondazione Policlinico Universitario A. Gemelli, IRCCS, Rome, Italy

[3] Department of Oncology and Hematology, Cytofluorimetry Unit, Central Hospital of Belcolle, Viterbo, Italy

[4] Department of Oncology and Hematology, Microbiology and Virology Unit, Central Hospital of Belcolle, Viterbo, Italy

*** Corresponding author**

Dr. Enzo M. Ruggeri, Department of Oncology and Hematology, Medical Oncology Unit, Central Hospital of Belcolle, Strada Sammartinese snc, 01100 Viterbo, Italy, Phone +390761339043, Fax +390761339039, e-mail: enzo.ruggeri@asl.vt.it, ORCID iD: 0000-0001-7790-0056

Supplementary Figure 1. Gating strategy for flow cytometry assay

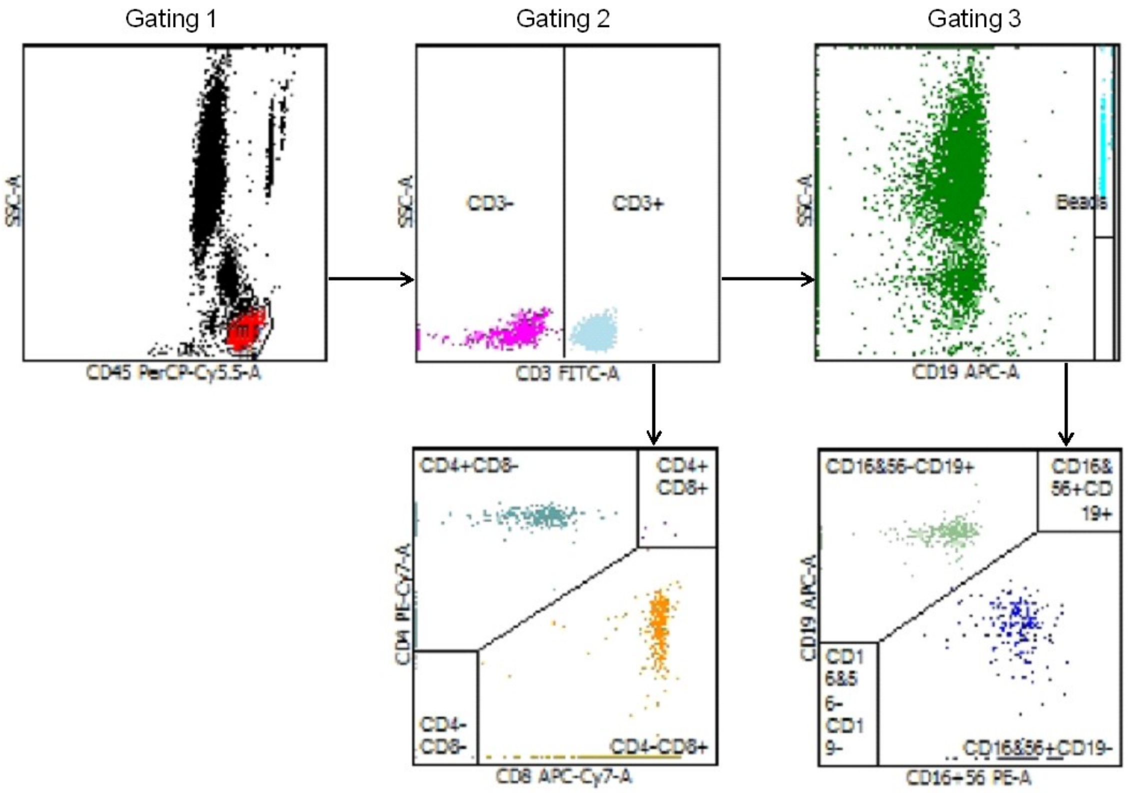

Gating 1: first gating on a plot of forward scatter (CD45^{+}) and side scatter (SSC-A) to detect the absolute value of lymphocytes; Gating 2: second gating on plot a forward scatter (CD3^{+}) and side scatter (SSC-A) to detect the absolute value of T lymphocytes and then gated on the CD8^{+} and CD4^{+} to detect the absolute values of T helper cells and T cytotoxic cells; Gating 3: third gating on a plot of forward scatter (CD19^{+}) and side scatter (SSC-A) to detect the absolute value of B lymphocytes and then gated on the CD16^{+}CD56^{+} and CD19^{+} to detect the absolute values of NK cells.

Supplementary Figure 2. Patients' flow diagram

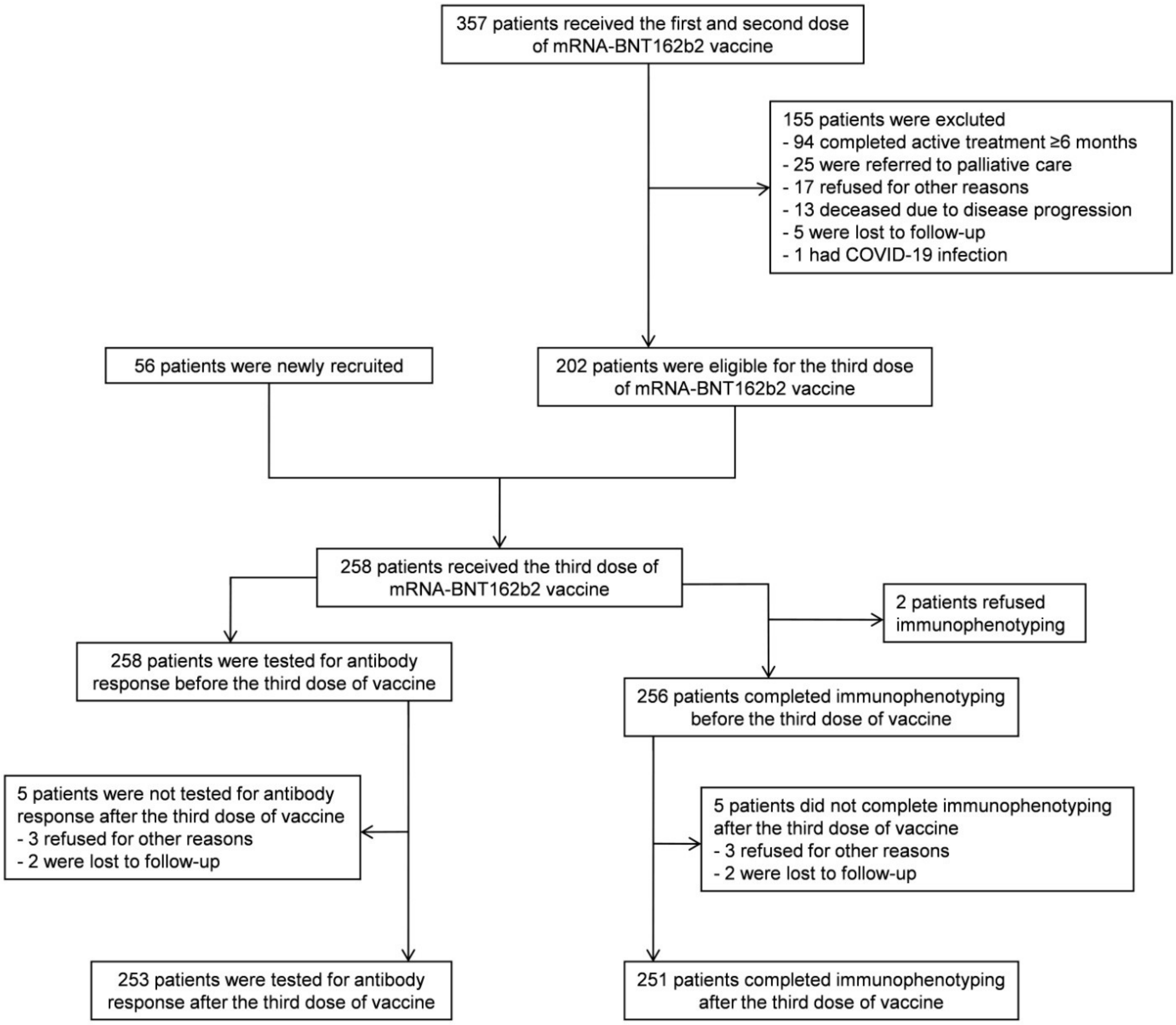

Supplementary Figure 3. Dynamic changes in peripheral lymphocyte subpopulation counts by type of active treatment.

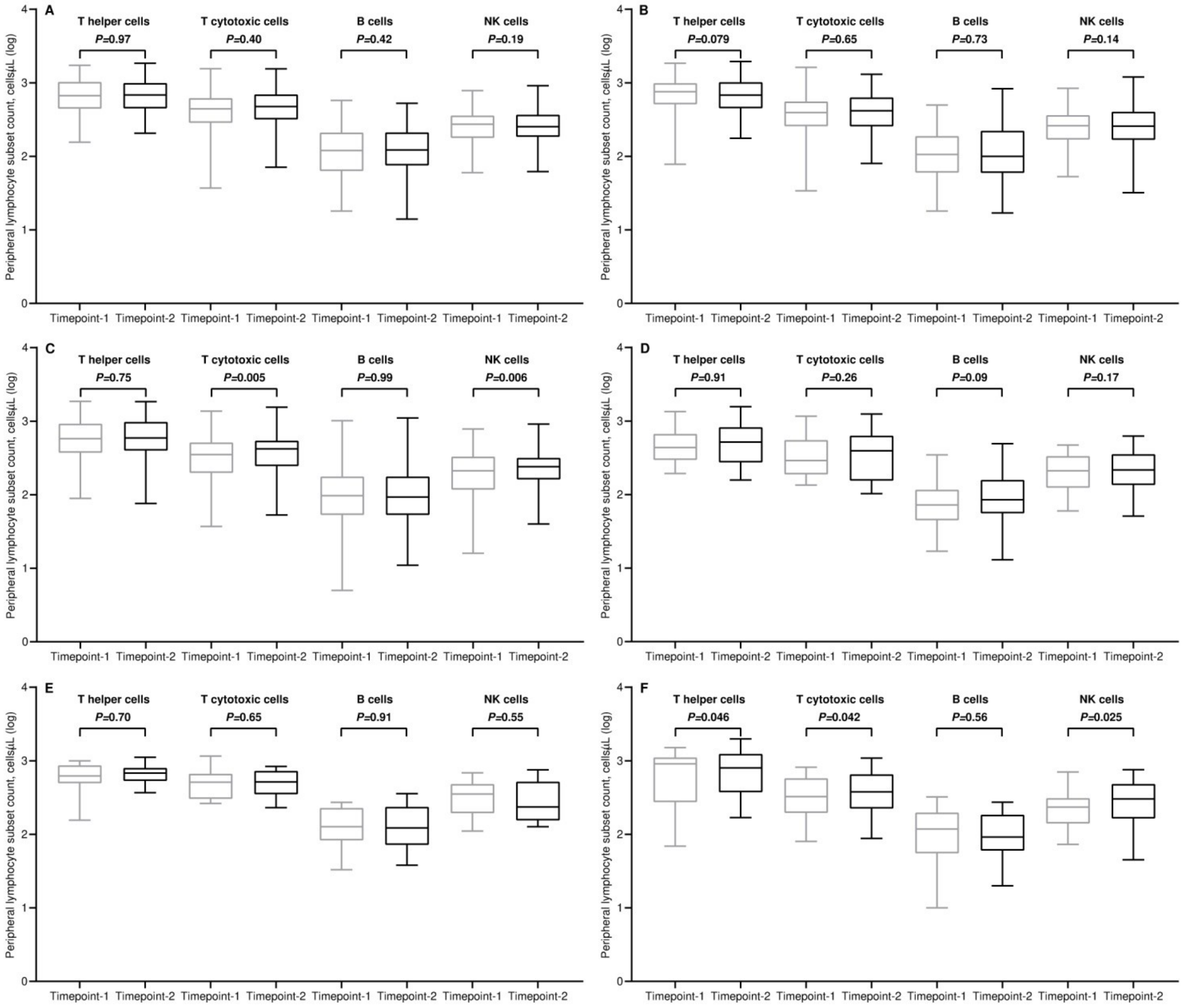

(A) Active surveillance. (B) Targeted therapies. (C) Cytotoxic chemotherapy. (D) Immune checkpoint inhibitors. (E) Hormonal therapies. (F) Cytotoxic chemotherapy and biological agents. Bars represent median values with 95% confidence intervals. Differences between groups were assessed using the Wilcoxon signed-rank test. A two-sided *P* value of <0.05 was considered statistically significant. Log, logarithmic; T helper cells, CD3+CD4+ cells; T cytotoxic cell, CD3+CD8+; B cells, CD19+; NK, Natural killer, CD16+CD56+. Timepoint-1 denotes assessment before the third dose of tozinameran; Timepoint-2 denotes assessment four weeks after the third dose of tozinameran.

Supplementary Figure 4. Dynamic changes in peripheral lymphocyte subpopulation counts by corticosteroid treatment.

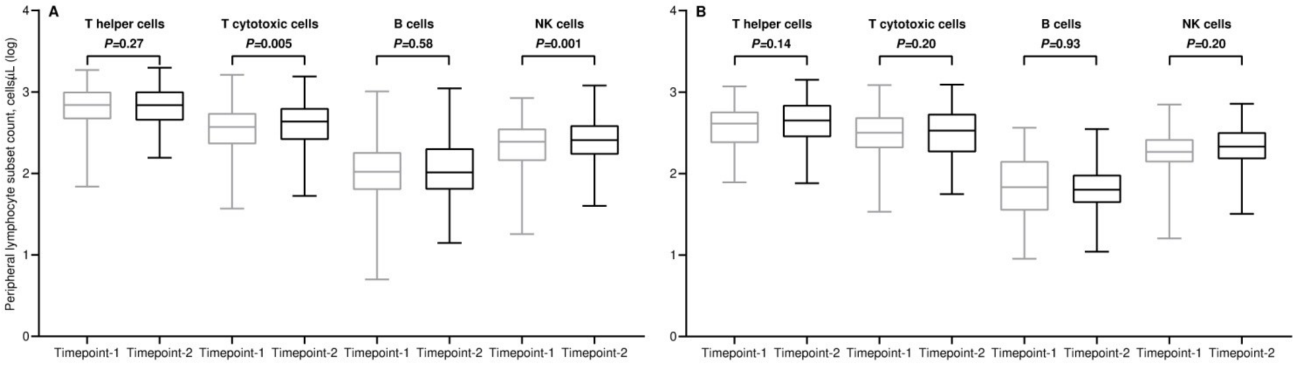

(A) No immunosuppressive corticosteroid therapy. (B) Immunosuppressive corticosteroid therapy with ≥10 mg prednisone equivalent daily for at least 7 days in the 28 days preceding the third dose of tozinameran. Bars represent median values with 95% confidence intervals. Differences between groups were assessed using the Wilcoxon signed-rank test. A two-sided *P* value of <0.05 was considered statistically significant. Log, logarithmic; T helper cells, CD3+CD4+ cells; T cytotoxic cell, CD3+CD8+; B cells, CD19+; NK, Natural killer, CD16+CD56+. Timepoint-1 denotes assessment before the third dose of tozinameran; Timepoint-2 denotes assessment four weeks after the third dose of tozinameran.

Supplementary Figure 5. Correlation between antibody titers and lymphocyte subpopulation counts in patients on active surveillance

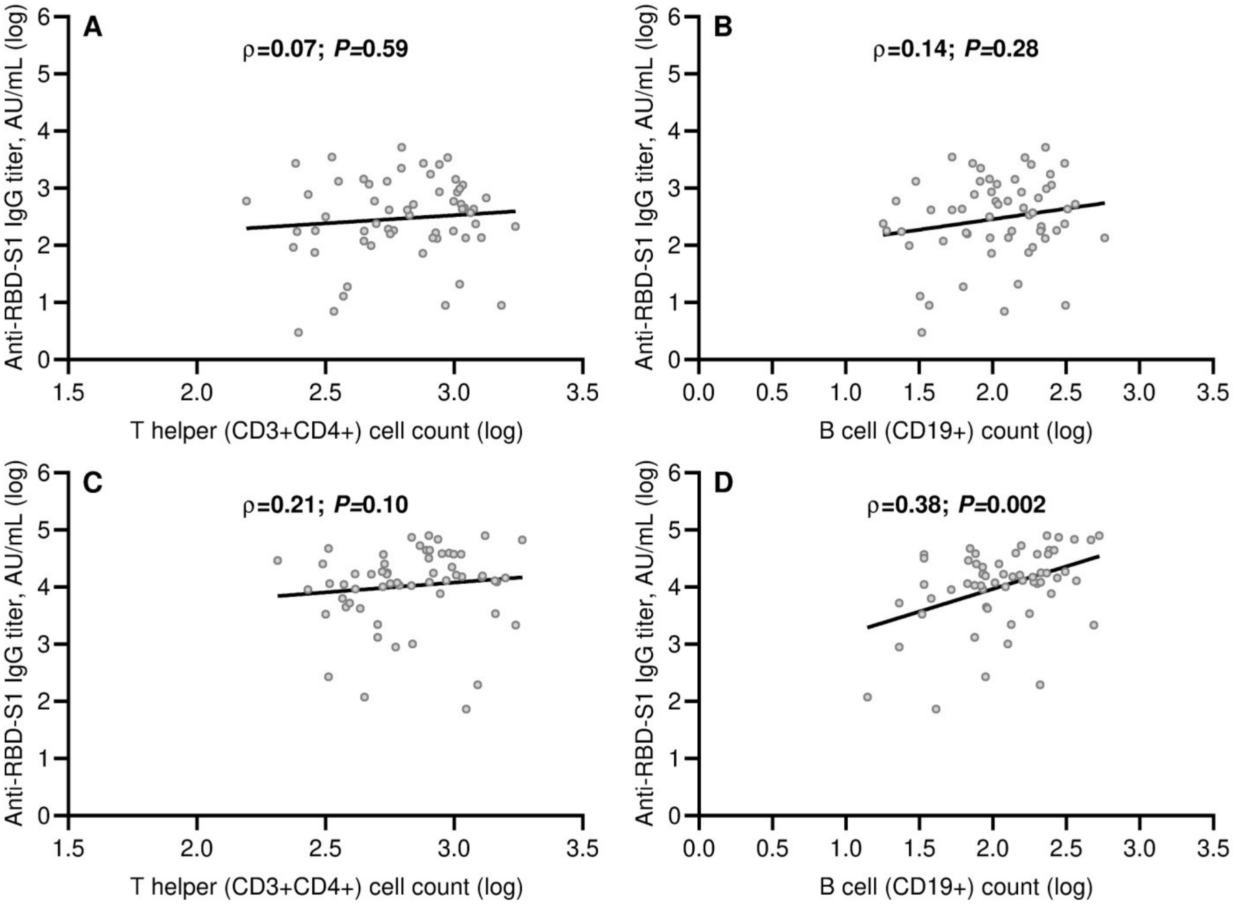

(A) T helper cell (CD3+CD4+) counts before the third dose of tozinameran. (B) B cell (CD19+) counts before the third dose of tozinameran. (C) T helper cell counts after the third dose of tozinameran. (D) B cell counts after the third dose of tozinameran. Correlation was assessed with the Spearman's test; a two-sided *P* value <0.05 was considered statistically significant. RBD-S1, receptor-binding domain (RBD) of the SARS-CoV-2 Spike protein (S1); AU, Arbitrary Unit; log, logarithmic values.

Supplementary Figure 6. Correlation between antibody titers and lymphocyte subpopulation counts in patients receiving targeted therapies

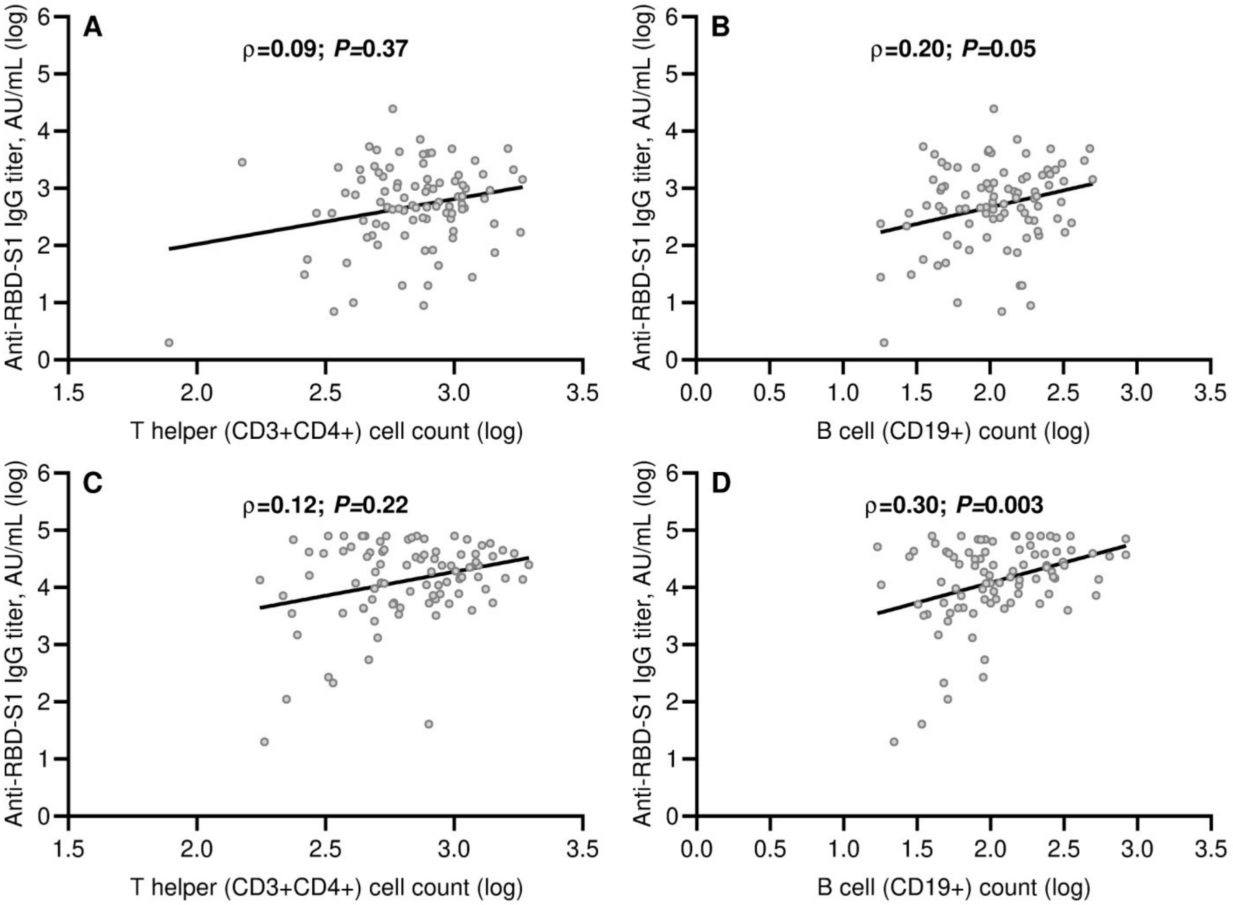

(A) T helper cell (CD3+CD4+) counts before the third dose of tozinameran. (B) B cell (CD19+) counts before the third dose of tozinameran. (C) T helper cell counts after the third dose of tozinameran. (D) B cell counts after the third dose of tozinameran. Correlation was assessed with the Spearman's test; a two-sided *P* value <0.05 was considered statistically significant. RBD-S1, receptor-binding domain (RBD) of the SARS-CoV-2 Spike protein (S1); AU, Arbitrary Unit; log, logarithmic values.

Supplementary Figure 7. Correlation between antibody titers and lymphocyte subpopulation counts in patients receiving cytotoxic chemotherapy

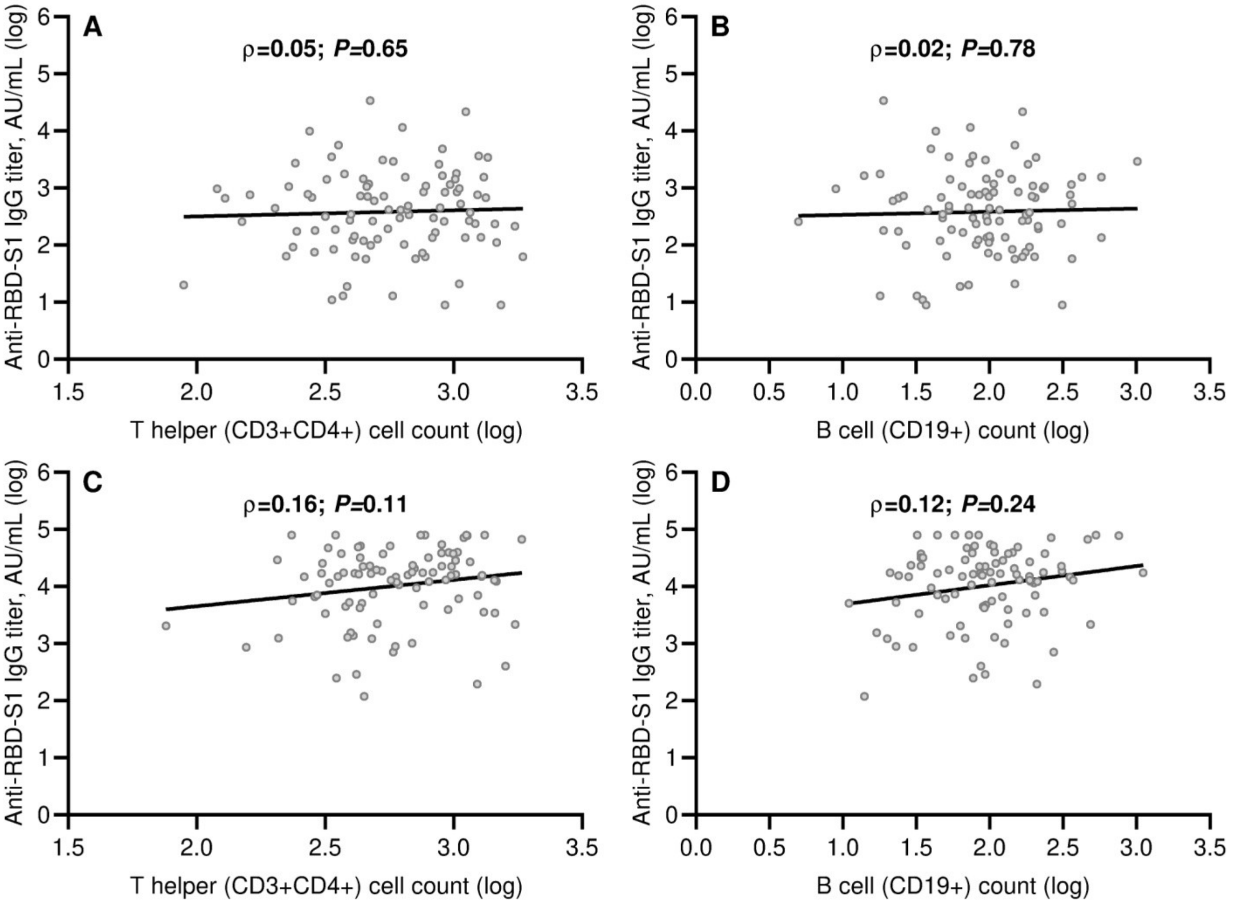

(A) T helper cell (CD3+CD4+) counts before the third dose of tozinameran. (B) B cell (CD19+) counts before the third dose of tozinameran. (C) T helper cell counts after the third dose of tozinameran. (D) B cell counts after the third dose of tozinameran. Correlation was assessed with the Spearman's test; a two-sided *P* value <0.05 was considered statistically significant. RBD-S1, receptor-binding domain (RBD) of the SARS-CoV-2 Spike protein (S1); AU, Arbitrary Unit; log, logarithmic values.

Supplementary Figure 8. Correlation between antibody titers and lymphocyte subpopulation counts in patients receiving immune checkpoint inhibitors

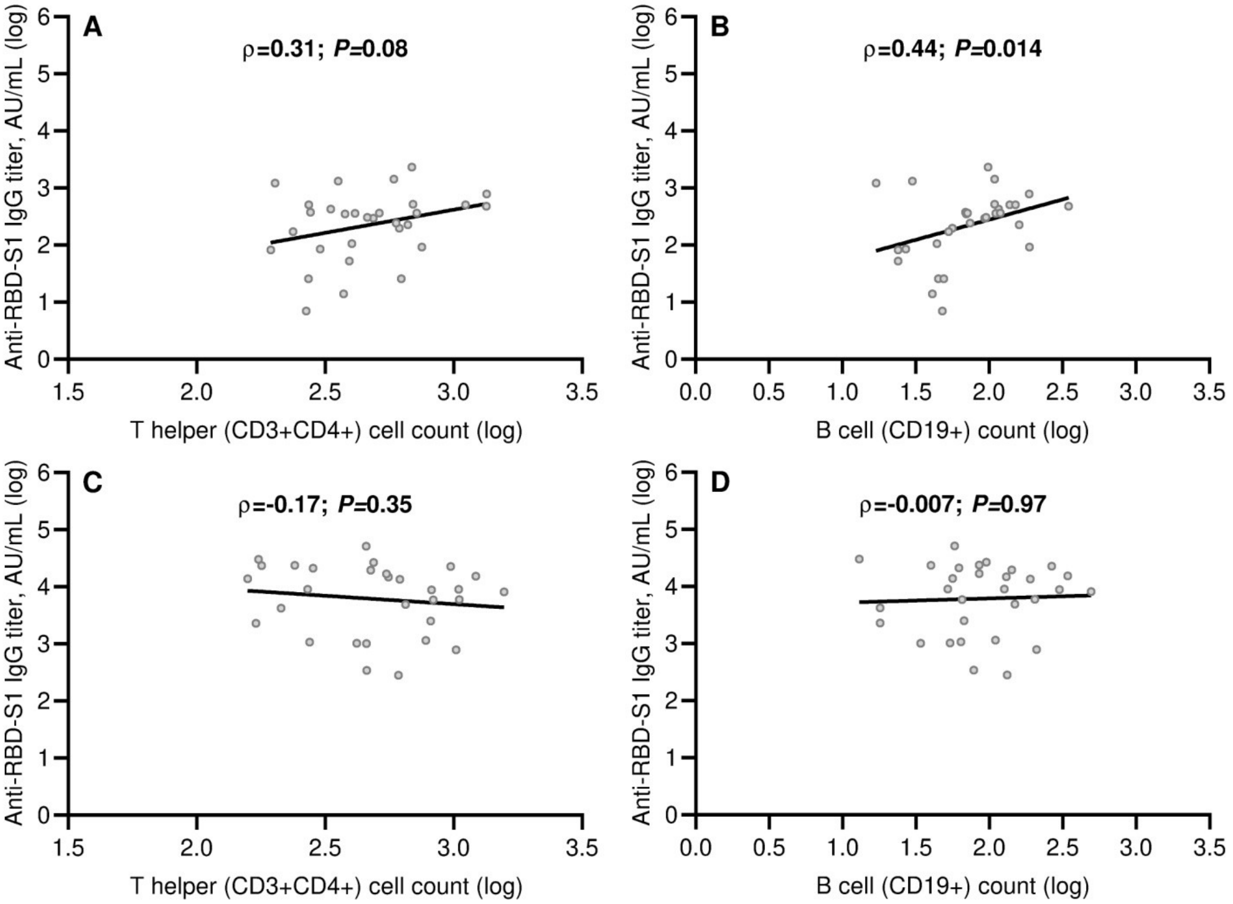

(A) T helper cell (CD3+CD4+) counts before the third dose of tozinameran. (B) B cell (CD19+) counts before the third dose of tozinameran. (C) T helper cell counts after the third dose of tozinameran. (D) B cell counts after the third dose of tozinameran. Correlation was assessed with the Spearman's test; a two-sided *P* value <0.05 was considered statistically significant. RBD-S1, receptor-binding domain (RBD) of the SARS-CoV-2 Spike protein (S1); AU, Arbitrary Unit; log, logarithmic values.

Supplementary Figure 9. Correlation between antibody titers and lymphocyte subpopulation counts in patients receiving hormonal therapies

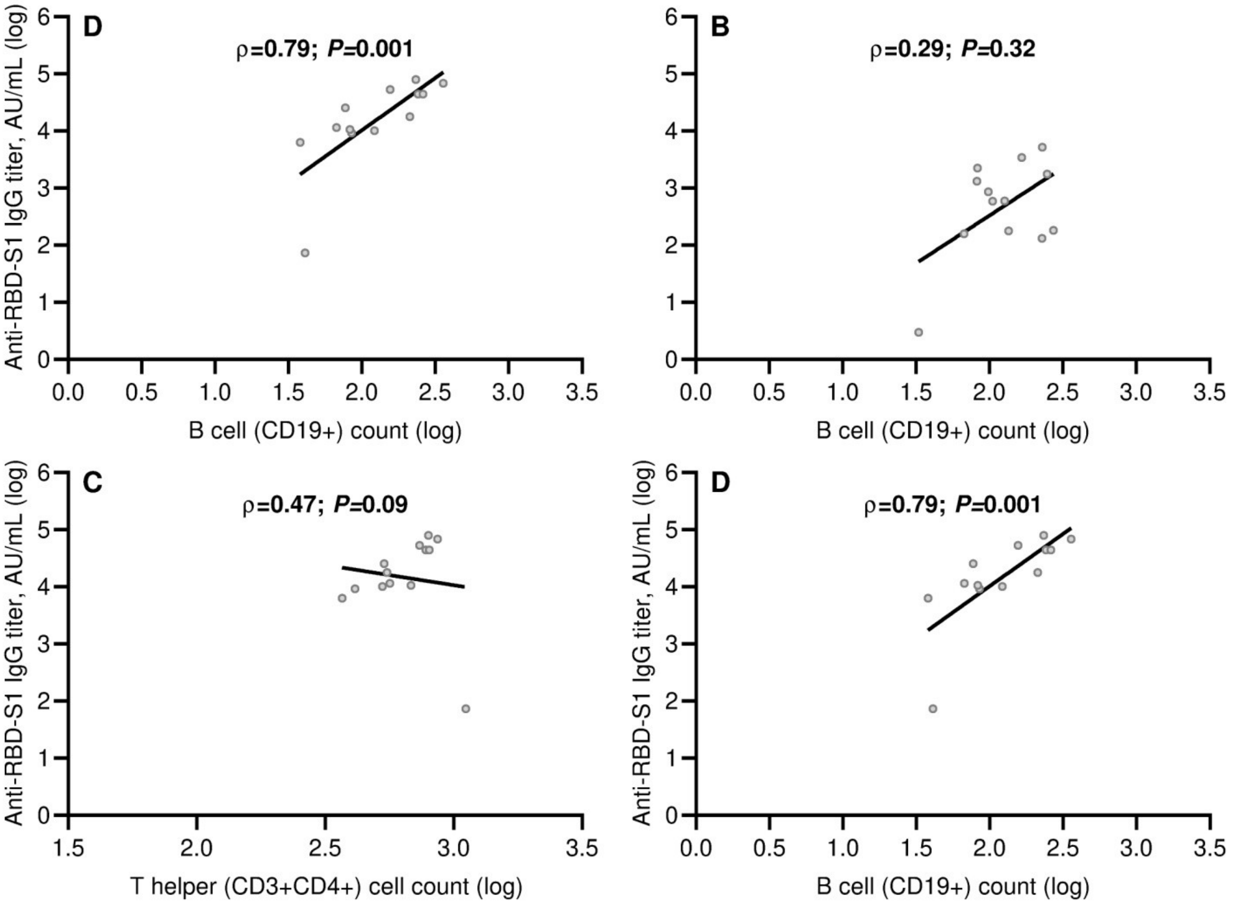

(A) T helper cell (CD3+CD4+) counts before the third dose of tozinameran. (B) B cell (CD19+) counts before the third dose of tozinameran. (C) T helper cell counts after the third dose of tozinameran. (D) B cell counts after the third dose of tozinameran. Correlation was assessed with the Spearman's test; a two-sided *P* value <0.05 was considered statistically significant. RBD-S1, receptor-binding domain (RBD) of the SARS-CoV-2 Spike protein (S1); AU, Arbitrary Unit; log, logarithmic values.

Supplementary Figure 10. Correlation between antibody titers and lymphocyte subpopulation counts in patients receiving combinations of cytotoxic chemotherapy and biological agents

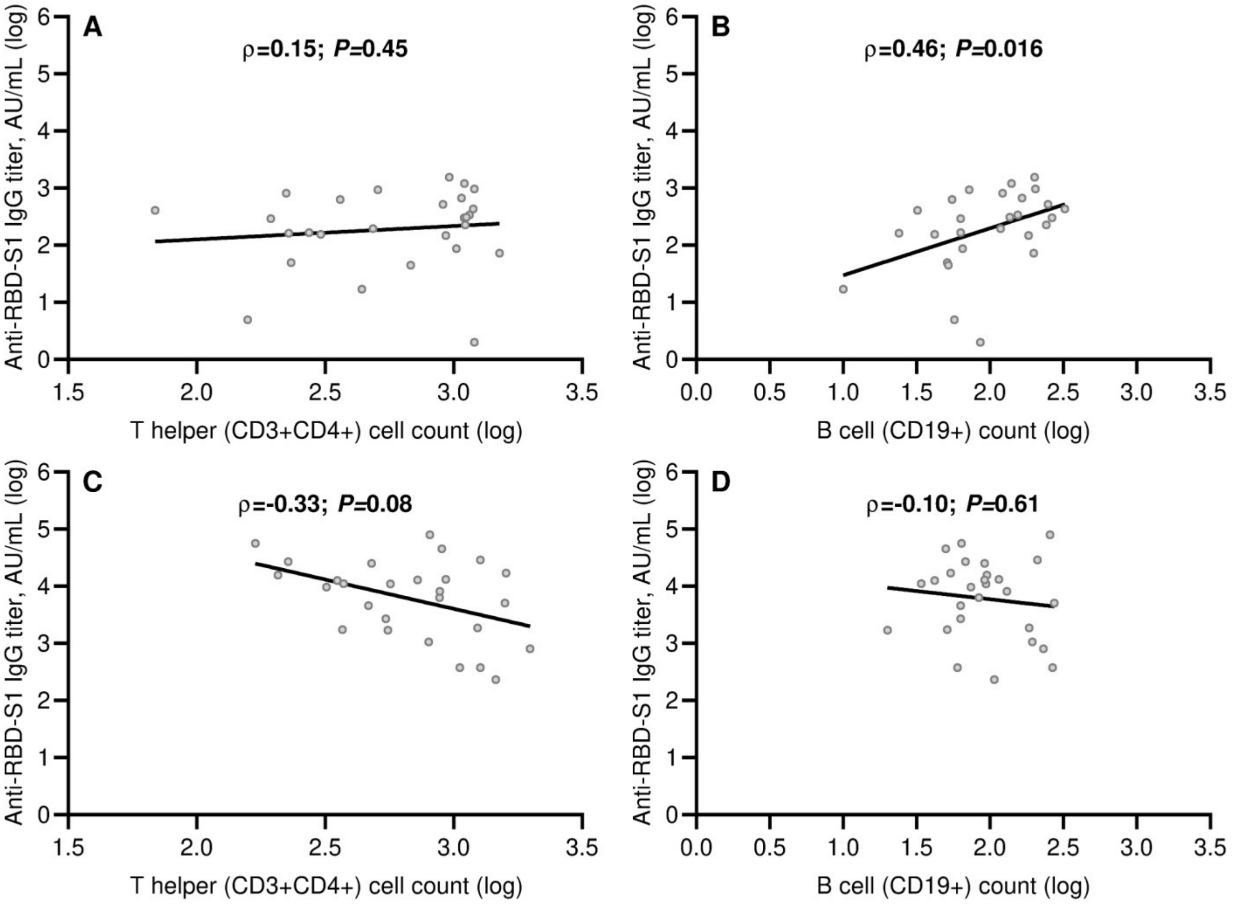

(A) T helper cell (CD3+CD4+) counts before the third dose of tozinameran. (B) B cell (CD19+) counts before the third dose of tozinameran. (C) T helper cell counts after the third dose of tozinameran. (D) B cell counts after the third dose of tozinameran. Correlation was assessed with the Spearman's test; a two-sided *P* value <0.05 was considered statistically significant. RBD-S1, receptor-binding domain (RBD) of the SARS-CoV-2 Spike protein (S1); AU, Arbitrary Unit; log, logarithmic values.

Supplementary Table 1. Multivariate analysis of peripheral lymphocyte counts by predefined clinical variables at timepoint-1.

Covariate	T helper cell count (log)		T cytotoxic cell count (log)		B cell count (log)		NK cell count (log)	
	Beta (95% CI)	P value	Beta (95% CI)	P value	Beta (95% CI)	P value	Beta (95% CI)	P value
Sex								
- male vs female	-0.02 (-0.09 to 0.03)	0.40	-0.03 (-0.11 to 0.04)	0.35	-0.02 (-0.12 to 0.07)	0.62	0.02 (-0.05 to 0.09)	0.67
Age (years)								
- >55 vs ≤55	0.05 (-0.02 to 0.12)	0.18	0.02 (-0.06 to 0.11)	0.54	-0.01 (-0.11 to 0.10)	0.93	0.13 (0.04 to 0.21)	**0.002**
ECOG PS								
- 0[a]	-	-	-	-	-	-	-	-
- 1	0.03 (-0.03 to 0.10)	0.33	-0.04 (-0.12 to 0.03)	0.26	0.01 (-0.08 to 0.11)	0.78	0.01 (-0.06 to 0.08)	0.86
- 2	-0.02 (-0.16 to 0.12)	0.76	0.01 (-0.14 to 0.18)	0.83	0.05 (-0.15 to 0.25)	0.63	-0.06 (-0.22 to 0.09)	0.43
Treatment setting								
- adjuvant or neoadjuvant[a]	-	-	-	-	-	-	-	-
- metastatic, first line	-0.09 (-0.16 to -0.01)	**0.023**	-0.09 (-0.18 to -0.01)	**0.032**	-0.08 (-0.20 to 0.02)	0.13	-0.07 (-0.16 to 0.01)	0.10
- metastatic, second or later line	-0.12 (-0.22 to -0.03)	**0.007**	-0.10 (-0.20 to 0.01)	0.06	0.02 (-0.13 to 0.13)	0.97	0.02 (-0.07 to 0.13)	0.61
Corticosteroid therapy[a]								
- yes vs no	-0.21 (-0.29 to -0.13)	**<0.001**	-0.03 (-0.12 to 0.06)	0.54	-0.14 (-0.26 to -0.02)	**0.017**	-0.04 (-0.14 to 0.04)	0.31
Type of active treatment								
- active surveillance[a]	-	-	-	-	-	-	-	-
- cytotoxic chemotherapy	-0.12 (-0.40 to 0.15)	0.38	-0.20 (-0.52 to 0.12)	0.22	-0.15 (-0.56 to 0.25)	0.44	-0.10 (-0.41 to 0.21)	0.52
- targeted therapy	-0.01 (-0.28 to 0.27)	0.96	-0.05 (-0.37 to 0.26)	0.74	-0.08 (-0.49 to 0.32)	0.68	0.02 (-0.29 to 0.33)	0.88
- immune checkpoint inhibitors	-0.15 (-0.44 to 0.13)	0.28	-0.10 (-0.43 to 0.22)	0.54	-0.23 (-0.65 to 0.18)	0.28	-0.05 (-0.38 to 0.26)	0.72
- hormonal therapy	-0.01 (-0.16 to 0.15)	0.97	0.01 (-0.15 to 0.18)	0.86	0.28 (0.06 to 0.49)	0.01	0.05 (-0.12 to 0.22)	0.57
- chemotherapy & biologics	-0.10 (-0.39 to 0.18)	0.48	-0.14 (-0.47 to 0.18)	0.39	-0.10 (-0.52 to 0.31)	0.64	-0.03 (-0.35 to 0.29)	0.84
Time from last active treatment								
- ≤28 days vs >28 days	0.09 (-0.19 to 0.37)	0.53	0.07 (-0.25 to 0.39)	0.66	0.10 (-0.30 to 0.51)	0.61	-0.01 (-0.33 to 0.29)	0.91

Statistically significant P values are highlighted in bold. *P* values derived from parametric 2-sided Wald's $\chi 2$ test with Bonferroni (α =0.01) correction for multiple comparisons. A two-sided *P* value of <0.05 was considered statistically significant. [a] Reference category. Log, logarithmic values; CI, confidence intervals; ECOG PS, Eastern Cooperative Oncology Group Performance Status. Corticosteroid therapy indicates ≥10 mg prednisone equivalent daily for at least 7 days in the 28 days preceding the third dose of vaccine; T helper cells, CD3+CD4+ cells; T cytotoxic cell, CD3+CD8+; B cells, CD19+; NK, Natural killer, CD16+CD56+; Timepoint-1 denotes assessment before the third dose of tozinameran.
